# A Preliminary Observation of Weight Loss Following Left Gastric Artery Embolization in Humans

**DOI:** 10.1155/2014/185349

**Published:** 2014-09-30

**Authors:** Andrew J. Gunn, Rahmi Oklu

**Affiliations:** ^1^Department of Imaging, Massachusetts General Hospital, Harvard Medical School, 55 Fruit Street, FND 216, Boston, MA 02114, USA; ^2^Division of Interventional Radiology, Department of Imaging, Massachusetts General Hospital, Harvard Medical School, 55 Fruit Street, 290 Gray/Bigelow, Boston, MA 02114, USA

## Abstract

*Background/Objectives*. Embolization of the left gastric artery (LGA), which preferentially supplies the gastric fundus, has been shown to produce weight loss in animal models. However, weight loss after LGA embolization in humans has not been previously established. The aim of this study was to evaluate postprocedural weight loss in patients following LGA embolization. *Subjects/Methods*. A retrospective analysis of the medical records of patients who underwent LGA embolization for upper gastrointestinal (GI) bleeding was performed. Postprocedural weight loss in this group was compared to a control group of patients who had undergone embolization of other arteries for upper GI bleeding. *Results*. The experimental group (*N* = 19) lost an average of 7.3% of their initial body weight within three months of LGA embolization, which was significantly greater than the 2% weight loss observed in the control group (*N* = 28) (*P* = 0.006). No significant differences were seen between the groups in preprocedural body mass index (BMI), age, postprocedural care in the intensive care unit, history of malignancy, serum creatinine, or left ventricular ejection fraction. *Conclusions*. The current data suggest that body weight in humans may be modulated via LGA embolization. Continued research is warranted with prospective studies to further investigate this phenomenon.

## 1. Introduction

The incidence of overweight and obese individuals in the United States has dramatically increased over the last twenty years and now includes nearly two-thirds of adults [1]. This results in significant morbidity and mortality often related to heart disease, stroke, type II diabetes, and even some cancers [1]. The current therapeutic alternatives for the overweight or obese patient include lifestyle modifications, a limited number of medical therapies, and bariatric surgery. However, the growing obesity epidemic would suggest that these methods alone are insufficient. Furthermore, invasive bariatric surgery carries a host of potential complications for the patient [2]. Recently, studies performed in animal models have demonstrated that body weight can be modulated via percutaneous, catheter-directed, transarterial embolization of the left gastric artery (LGA), the artery that preferentially provides blood flow to the fundus of the stomach [3–6], although this effect has not been established in humans. To further explore the possibility that a catheter-directed approach may provide a minimally invasive therapeutic alternative for obesity, we performed a single-center retrospective review of patients who underwent LGA embolization to determine whether this intervention led to weight loss.

## 2. Materials and Methods

This study was approved by our institutional review board (IRB) and is HIPAA-compliant. Given the retrospective nature of the evaluation, the requirement for obtaining informed consent was waived.

Hospital records were reviewed to identify all adult patients who underwent catheter-directed embolization of any branch of the celiac trunk for upper gastrointestinal (GI) bleeding from 2000 to 2012. Patients were required to have three separate weights recorded in the electronic medical record (EMR) which consisted of (1) preembolization body weight recorded within the four weeks prior to the procedure, (2) an early postembolization weight recorded within the first 3 months after the procedure, and (3) a delayed postembolization weight recorded at least 3 months after the procedure. The first recorded weight in the EMR within 3 months of the embolization procedure served as the early postembolization weight. Any change in subject weight recorded at this early time point is referred to as “immediate change.” The first weight in the EMR to be recorded at least 3 months after the embolization procedure served as the delayed postembolization weight. Changes in subject weight at this delayed time point are referred to as “delayed change.” Patients who underwent embolization of the LGA comprised the experimental group while patients who had an embolization of any other artery of the celiac trunk comprised the control group. Pertinent data were collected from the EMR with a focus on potential confounding factors for weight loss such as history of malignancy, chemotherapy during the study period, participation in any weight-loss programs during the study period, postprocedural intensive care unit (ICU), left ventricular ejection fraction (LVEF) as an indicator of congestive heart failure (CHF), and serum creatinine as a marker of chronic kidney disease (CKD). The body mass index (BMI) of each patient was also recorded at each time point. Changes in weight were expressed as a percentage of the original preembolization weight at the early time point (immediate change) and delayed time point (delayed change).

### 2.1. Statistical Analysis

Data were analyzed using GraphPad Prism 5.2 (La Jolla, CA) software utilizing Student's *t*-test with a *P* value <0.05 considered to be as significant.

## 3. Results

### 3.1. Patient Selection

By using the search term “left gastric artery embolization,” the database retrieved 1213 adult patients from 2000 to 2012. 797 of these patients had undergone a catheter-directed arterial embolization of a celiac trunk branch. Of these 797 patients, 40 patients had LGA embolization and 757 patients had another branch of the celiac trunk embolized. 599 patients (1 patient from the experimental group and 598 patients from the control group) were excluded from analysis because the embolization procedure was performed for reasons other than upper GI bleeding. An additional 151 patients (20 patients from the experimental group and 131 patients from the control group) were excluded from analysis because they did not have the three requisite body weights documented in the EMR. Thus, 19 patients were eligible for analysis in the experimental group while 28 patients were eligible from the control group.

### 3.2. Patient Characteristics

The experimental group comprised 12 males and 7 females with a mean age of 64.6 years (range: 46–92) and a mean BMI of 30.3 (range: 20.3–58.9). The control group comprised 15 males and 13 females with a mean age of 58.7 years (range: 22–82) and a mean BMI of 29.2 (range: 18.1–50.3). No difference in the mean age (*P* = 0.20) or preprocedural BMI (*P* = 0.70) was seen between the groups. 58% of the experimental group had a history of malignancy and four of these patients were receiving chemotherapy during the study period. 50% of the control group had a history of malignancy and six of these patients were receiving chemotherapy during the study period. The types of malignancies in the experimental group and in the control group are shown in Table 1. There was no significant difference in the proportion of patients with a history of malignancy (*P* = 0.29) or receiving chemotherapy during the study period (*P* = 0.97) between the two groups. Patients in the experimental group spent an average of 5.3 days (range: 2–8 days; median: 6 days) in the ICU after embolization, had a mean LVEF of 62% (range: 29–77%; median: 69%), and had a mean serum creatinine of 0.8 mg/dL (range: 0.5–1.7; median: 0.75). Patients in the control group spent an average of 13.1 days (range: 3–45 days; median: 6 days) in the ICU after embolization, had a mean LVEF of 68% (range: 56–75%; median: 69%), and had a mean serum creatinine of 0.9 mg/dL (range: 0.6–2.3; median: 0.8). No significant differences between the two groups were found in the ICU postprocedural care (*P* = 0.34), mean LVEF (*P* = 0.25), or mean serum creatinine (*P* = 0.48). No patients in either group participated in a formal weight-loss program during the study period.

The causes of upper GI bleeding in the experimental group and in the control group are shown in Table 2. The arteries embolized in the control group included splenic (*N* = 13), gastroduodenal (*N* = 7), right hepatic (*N* = 4), and left hepatic (*N* = 4) arteries. The method of arterial embolization in the experimental group included coils (*N* = 9), gelfoam (*N* = 5), and polyvinyl alcohol (PVA) particles (*N* = 5). The method of arterial embolization in the control group included coils (*N* = 23), gelfoam (*N* = 3), and PVA particles (*N* = 2). PVA particles ranging from 300 to 500 *μ*m, 500 to 710 *μ*m, and 710 to 1000 *µ*m were used, based on operator preferences.

### 3.3. Patient Weights

**Figure 1:**
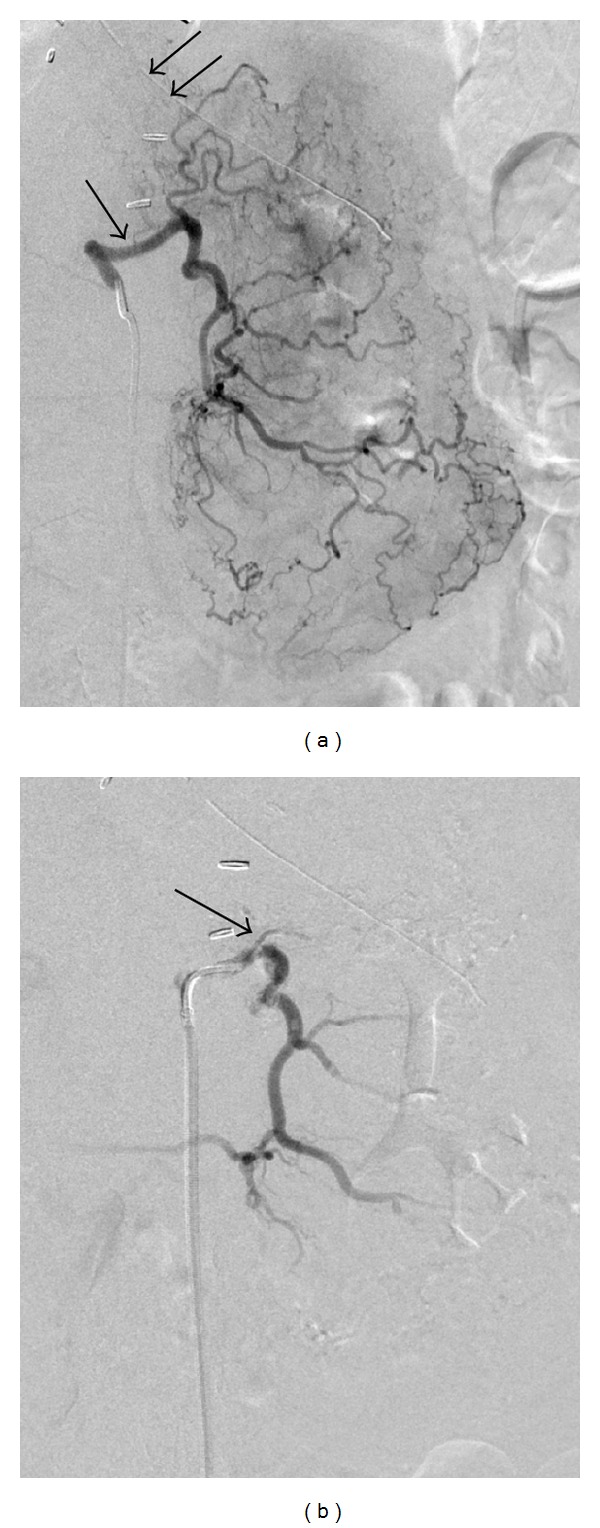
72-year-old male was found to have bleeding at the gastric fundus on endoscopy. (a) Digital subtraction angiography (DSA) of the LGA (black arrow) demonstrated no evidence of contrast extravasation to suggest bleeding. A nasogastric tube is also seen (double black arrows). (b) DSA performed after gelfoam embolization of the LGA revealed a significant decrease in the opacification of the arterial branches in the fundus (black arrow).

**Figure 2:**
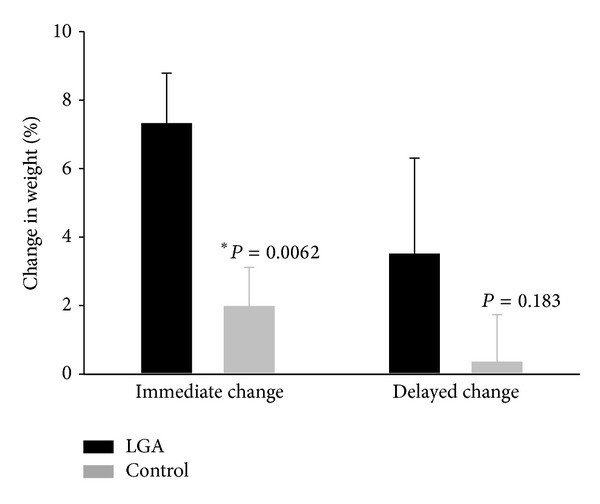
Bar graph comparing the change in weight between patients after LGA embolization (black bars) or embolization of another celiac trunk branch (gray bars). Patients lost significantly more weight after LGA embolization at the early time point (*P* = 0.006) but the difference in weight loss was not statistically significant at the delayed time point (*P* = 0.183).

The early postembolization weights were recorded at a mean of 1.6 months (range: 1–2.9, median: 1.3) in the experimental group and 1.5 months (range: 0.1–2.9, median: 1.6) in the control group (*P* = 0.70). The delayed postembolization weights were recorded at a mean of 13.6 months (range: 3–102.3 months, median: 10.4) in the experimental group and 4 months (range: 3–22.4 months, median: 4.5) in the control group (*P* = 0.007). Patients who underwent LGA embolization lost, on average, 7.3% of their body weight at the early time points (immediate change) (range: −1% to 17.9%, SEM: 1.4) (Figures 1 and 2). This was significantly greater than the 2% weight loss (range: −18.3% to 13.6%, SEM: 1.1) observed in the control group over the same period (*P* = 0.006). At the delayed time points, patients had lost an average of 3.5% of their preembolization body weight after LGA embolization (range: −21.1% to 24.3%, SEM: 1.4). Patients in the control group had lost only an average of 0.3% of their preembolization body weight at the delayed time points (range: −17% to 11.5%, SEM: 1.4), which was not statistically different from the experimental group (*P* = 0.18). Weight loss was similar for patients with a preprocedural BMI in the “obese” range (≥30) compared to patients with a BMI of <30 for both groups at both time points. Weight loss was also similar for patients who received chemotherapy during the study period in comparison to those who received no chemotherapy for both groups at both time points. When comparing weight loss in patients who underwent embolization using permanent embolic materials (for example, coils or PVA particles) compared to embolization using the nonpermanent gelfoam material, there was no significant difference in weight loss for either group at either time point.

## 4. Conclusions

Arepally et al. were the first to suggest a percutaneous, catheter-based approach to treat obesity [3]. Their hypothesis was that selective LGA embolization could cause relative ischemia in the mucosa of the gastric fundus, which could, in turn, suppress the production of the hormone ghrelin. Studies have shown that ghrelin, which is primarily secreted from the mucosa of the gastric fundus, has a powerful orexigenic effect, stimulating food intake and weight gain in both animal and human models [7–9]. Thus, there is the possibility that an intervention that acts to lower circulating ghrelin levels has potential to treat obesity. Indeed, multiple preclinical studies have been able to demonstrate suppressed serum ghrelin levels and weight modification after LGA embolization using both sodium morrhuate and clinically available beads as embolic agents [3–6]. In humans, however, weight loss after LGA embolization has not been reported; since celiac angiography and embolization of arterial branches arising from the celiac trunk is a routine practice in interventional radiology, we analyzed patients that had undergone LGA embolization to determine whether this intervention led to weight loss. Our data shows that patients undergoing LGA embolization to stop a bleeding vessel lost more weight within three months of embolization than a control group (*P* = 0.006) although there was no significant difference in the degree of weight loss between the two groups at the delayed time point (*P* = 0.18) (Figure 2). Of note, the “delayed change” weights of the experimental group were recorded in the EMR at a much later time point (mean: 13.6 months) than those of the control group (mean: 4 months) (*P* = 0.007). Prospective studies involving a more rigorous weight measurement design may reveal the degree of weight change potentially missed in this retrospective study between 4 months and 13.6 months.

The weight loss in our study was modest and less sustained when compared to that seen in bariatric surgery [10] which may be due to recanalization of the embolized LGA, the development of collateral flow to the fundus reestablishing ghrelin production, and/or compensatory ghrelin production from other sites in the body. Further, the goal of care in the groups of patients evaluated in this study was to stop a bleeding vessel and not to achieve weight loss. Therefore, it is hoped that future refinements of the procedure, including the use of liquid embolic agents, will be able to achieve greater and more sustained weight loss. Moreover, if effective, transarterial embolization of the LGA would undoubtedly provide obese patients with a much less morbid therapeutic option [2, 6].

Our study is limited by several factors. First, the analysis was retrospective in nature and thus we were unable to correlate our findings with patients' serum ghrelin levels or assess the efficacy of LGA in an exclusively obese patient population. Even though we attempted to provide matched controls, it would be impossible to control for the many other potentially confounding variables involved in weight loss. Second, the study was limited by a small sample size which was due, in large part, to the fact that 599 patients were excluded from analysis as their embolizations were performed for reasons other than upper GI bleeding. These excluded embolizations were most commonly done for the treatment of cancer via either chemoembolization or selective internal radiation therapy (SIRT). An additional 151 patients were excluded from analysis because they did not have the requisite weights recorded in the EMR. In spite of these limitations, the data suggest that transarterial embolization of the LGA has the potential to become a therapeutic option for the bariatric patient. Randomized, prospective studies are certainly needed to confirm its safety, efficacy in humans, and role in relation to bariatric surgery.

## Figures and Tables

**Table 1:** Number and types of malignancies in each patient group.

Experimental group (*N* = 11/19)	Control group (*N* = 14/28)
Colorectal (*N* = 3)	Colorectal (*N* = 2)
Lung (*N* = 1)	Pancreatic (*N* = 3)
Laryngeal (*N* = 1)	Laryngeal (*N* = 1)
Renal (*N* = 1)	Renal (*N* = 1)
Prostate (*N* = 2)	Prostate (*N* = 1)
Gastric (*N* = 2)	Lymphoma (*N* = 2)
Mesothelioma (*N* = 1)	Neuroendocrine (*N* = 1)
	Ovarian (*N* = 1)
	Endometrial (*N* = 1)
	Melanoma (*N* = 1)

**Table 2:** Causes for upper GI bleeding.

Experimental group (*N* = 19)	Control group (*N* = 28)
Gastric ulcers (*N* = 8)	Duodenal ulcers (*N* = 7)
Nasogastric or gastrostomy tube placement (*N* = 3)	Splenic artery pseudoaneurysm (*N* = 7)
Gastric varices (*N* = 3)	Surgical complication (*N* = 4)
Gastric cancer (*N* = 2)	Percutaneous complication∗ (*N* = 3)
Metastasis to the stomach (*N* = 1)	Pancreatitis (*N* = 2)
Pancreatitis (*N* = 1)	Hepatic artery pseudoaneurysm (*N* = 2)
Hematemesis in setting of anticoagulation (*N* = 1)	Pancreatic cancer (*N* = 1)
	Trauma (*N* = 1)
	Liver metastasis (*N* = 1)

*Indicates complications arising from either percutaneous biopsy or percutaneous abscess drainage.
